# UFO: a web server for ultra-fast functional profiling of whole genome protein sequences

**DOI:** 10.1186/1471-2164-10-409

**Published:** 2009-09-02

**Authors:** Peter Meinicke

**Affiliations:** 1 Department of Bioinformatics, Institute of Microbiology and Genetics, Georg-August-University Göttingen, Germany

## Abstract

**Background:**

Functional profiling is a key technique to characterize and compare the functional potential of entire genomes. The estimation of profiles according to an assignment of sequences to functional categories is a computationally expensive task because it requires the comparison of all protein sequences from a genome with a usually large database of annotated sequences or sequence families.

**Description:**

Based on machine learning techniques for Pfam domain detection, the UFO web server for ultra-fast functional profiling allows researchers to process large protein sequence collections instantaneously. Besides the frequencies of Pfam and GO categories, the user also obtains the sequence specific assignments to Pfam domain families. In addition, a comparison with existing genomes provides dissimilarity scores with respect to 821 reference proteomes. Considering the underlying UFO domain detection, the results on 206 test genomes indicate a high sensitivity of the approach. In comparison with current state-of-the-art HMMs, the runtime measurements show a considerable speed up in the range of four orders of magnitude. For an average size prokaryotic genome, the computation of a functional profile together with its comparison typically requires about 10 seconds of processing time.

**Conclusion:**

For the first time the UFO web server makes it possible to get a quick overview on the functional inventory of newly sequenced organisms. The genome scale comparison with a large number of precomputed profiles allows a first guess about functionally related organisms. The service is freely available and does not require user registration or specification of a valid email address.

## Background

The assignment of genes to certain functional categories is a central task in genome annotation. The distribution of assignments, i.e. the functional profile, provides a highly informative summary of a genome. Functional profiling plays a key role in comparative genomics for studying aspects of systems biology on a genome wide scale [1]. Without the restriction of DNA sequencing to culturable organisms, metagenomics allows to study the genomic potential of whole microbial communities. Functional profiling of metagenomes is an essential tool for comparative analysis of microbial ecosystems [2]. In the context of functional genomics, gene clusters and protein domains are widely used for homology-based annotation. Both approaches cover different aspects of the annotation and are often used in parallel to obtain a comprehensive description. While gene clusters as used for COGs [3] or within the SEED framework [4] provide a valuable resource for functional annotation based on the identification of homologous genes, the domain based approach is focussed on modelling and detection of functional modules which usually involve only parts of a gene. At the level of functional modules, the Pfam domain family database [5] currently provides the highest coverage. State-of-the-art methods for protein domain detection, like HMMER [6], are computationally expensive and several approximation techniques have been suggested to accelerate the model based prediction of protein domains. With a slight loss of sensitivity fast prefiltering methods can achieve speed ups of about two orders of magnitude as compared with HMMER [7]. Computational speed is of particular importance for the design of web-based sequence analysis tools. Due to computational expense most web servers for protein domain search only provide a single-sequence submission interface [8,9]. In addition to single sequence submission, the Pfam web server [5] also offers a batch option which allows the user to submit small multiple fasta files. These files are restricted to a maximum of 1000 protein sequences with a maximum sequence length of 2000 residues.

Using machine learning techniques for feature-based protein sequence classification [10-12], the UFO web server for **u**ltra-fast **f**unctional pr**o**filing provides an instantaneous estimation of Pfam profiles, i.e. frequencies of Pfam domains, for large sets of protein sequences. With a speed up of four orders of magnitude, UFO is well prepared to cope with the rapidly growing amount of genomic and metagenomic sequence data.

## Construction and content

The UFO web server has been built around an efficient implementation of machine learning techniques for protein sequence classification which have been described in [10-12]. Fast feature-based techniques for protein sequence representation have been combined with a multi-class multi-label approach [12] to assign protein sequences to Pfam domain families. While our previous model was obtained from training with about 1.5 × 10^5 ^sequences from the Pfam A release 22 seed alignments, UFO is based on training with the complete Pfam A release 23 full alignments which comprise more than 6 × 10^6 ^domain sequences. As an important difference, our previous publication [12] only considers a prefiltering method that uses the family specific scores from feature space discriminants to produce a ranking of domain models which in turn can be used to reduce the set of HMMER models in subsequent searches. UFO also uses a high-dimensional word-based feature space [11] according to a word length of 20 amino acids, but in addition the discriminant scores of the five highest scoring domain families are passed to a small neural network to decide whether a score actually indicates a valid match. The neural network architecture and its training has been described in [13] for the case of metagenomic gene prediction. UFO uses a network with five hidden units and with three inputs which correspond to the particular discriminant score and the mean and maximum score over all models. The output corresponds to an estimated posterior probability of a true match. Currently, domain families with a probability above 0.5 are reported as valid matches. In comparison with profile hidden Markov models [6], the feature-based machine learning approach does not provide a localization of protein domains but merely an indication of the presence or absence of a certain domain within a protein sequence. This implies that also the order or the repetition of domains cannot be predicted by the utilized approach. However, for the purpose of functional profiling this kind of "pure" domain detection usually does not mean a limitation. Actually, it has been shown that the prediction of protein function can be realized fairly well without considering domain repetitions or the ordering of domains [14]. For reasons of speed, another restriction as compared with Pfam/HMMER arises from the maximum number of domain families which can be detected within a single sequence. Currently, a protein sequence can be assigned to at most five different families. Only in rare cases we observed that this number was exceeded in the existing annotations.

In the first instance, the UFO server provides an ultra-fast search engine for detection of protein domains [5] according to the Pfam A release 23 from July 2008 which comprises 10340 domain families. In addition, UFO contains the precomputed profiles of 821 genomes from the HAMAP database (release from March 2009) [15] which are used for profile comparison. These reference genomes include 54 archaeal, 721 bacterial, and 46 eukaryotic proteomes, respectively. The complete list of reference genomes can be found in one of the UFO output files which are described in the next section. As a dissimilarity measure UFO utilizes the "profile divergence" with respect to these proteomes, which is computed in terms of Jeffreys' J-divergence, a symmetrized version of the Kullback-Leibler divergence between two probability distributions [16]. Given two profiles *P, Q *with estimated domain probabilities *p*_*i*_, *q*_*i *_for *n *domain families the profile divergence is

(1)

The probabilities are estimated from the corresponding domain frequencies using a pseudocount parameter *c*. A suitable value of *c *was determined by hierarchical cluster analysis based on the above divergence measure. For that purpose, a complete linkage clustering was applied to a collection of 1017 prokaryotic profiles from 21 different phyla. To cope with the typical database bias towards particular culturable organisms [17], from all profiles that correspond to the same genus only the medoid profile, which by definition yields the minimal sum of divergences to the members of that genus, was selected for clustering. For a varying pseudocount parameter with 101 logarithmically spaced values in the interval [10^-8^, 10^2^] and each partition in the range between 10 and 50 clusters the agreement of the clustering with the given taxonomic groups on phylum level was measured by the adjusted Rand index [18]. The best agreement was obtained for a pseudo count *c *= 0.01 with 22 clusters which resulted in a maximal adjusted Rand index of 5.17. For that partition the maximal within cluster divergence was *d*_*c *_= 3.53. This value is actually used by the UFO server to scale the profile divergence by *D*(*P, Q*)/*d*_*c *_to a more meaningful range, where values clearly below 1 usually correspond to phylogenetically and functionally related organisms.

## Utility and discussion

### User interface

**Figure 1:**
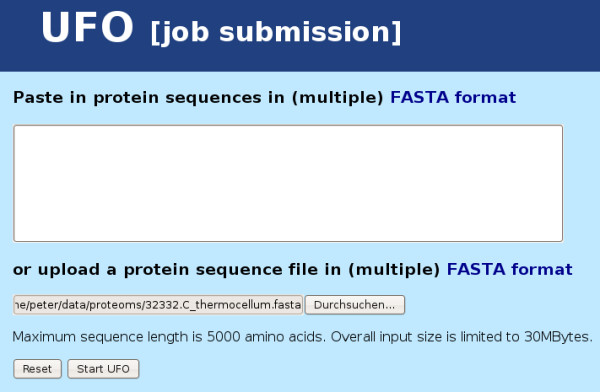
**Screenshot: UFO job submission**. Screenshot of the UFO job submission page.

**Figure 2:**
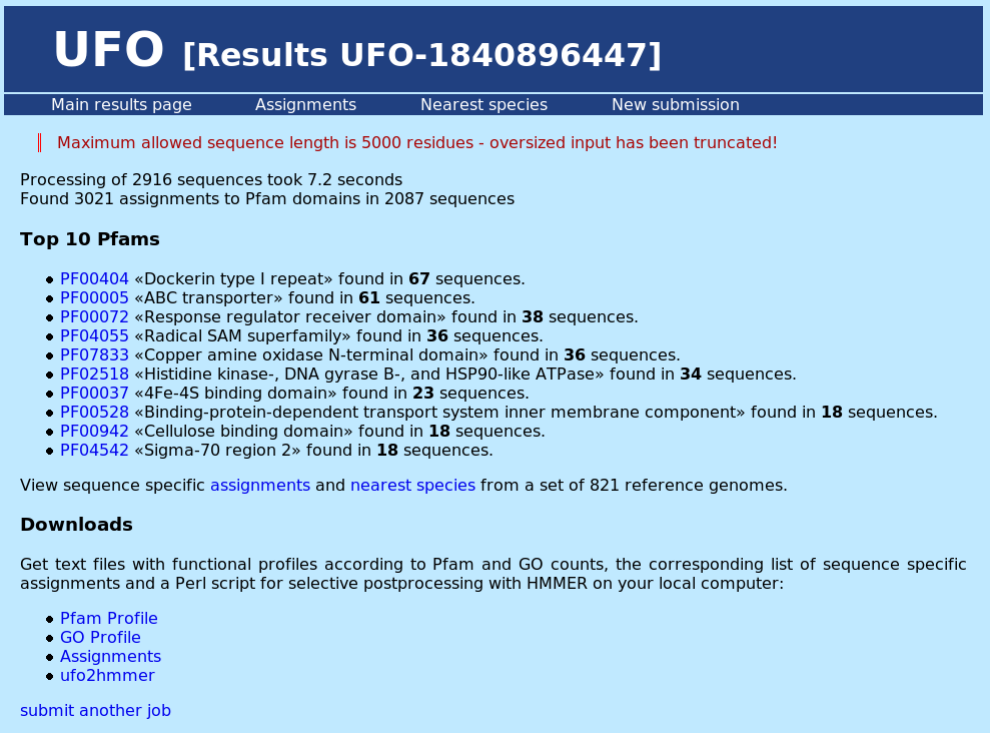
**Screenshot: UFO example results**. Screenshot of an UFO example "main results" page based on *C. thermocellum *(strain DSM 4150) proteome.

**Figure 3:**
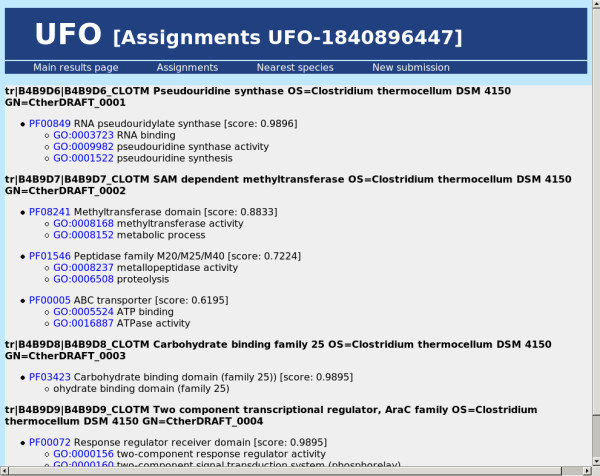
**Screenshot: UFO assignments**. Screenshot of an UFO example "assignments" page based on *C. thermocellum *(strain DSM 4150) proteome.

**Figure 4:**
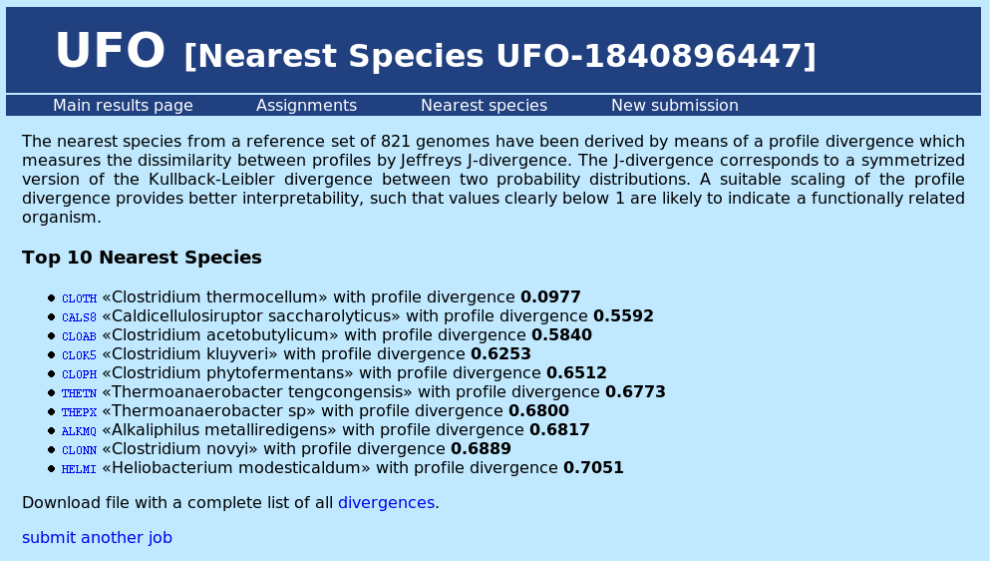
**Screenshot: UFO nearest species**. Screenshot of an UFO example "nearest species" page based on *C. thermocellum *(strain DSM 4150) proteome.

Considering the functionality of the UFO web server application, its use proceeds in the following manner: first, the user submits a collection of protein sequences in multiple fasta format either by pasting into the sequence input window or by uploading a valid multiple fasta file (see Figure 1). The maximum overall input size is 30 Mbyte. Sequences longer than 5000 amino acids are truncated and a corresponding warning is displayed. After submission the status of the processing is displayed in intervals of two seconds until the output files have been written. Then the results are shown on a new page which provides several statistics, links for additional information, and a download section (see Figure 2). The statistics comprise runtime, the total number of detected domains and a top ten list of the most abundant domain families together with hyperlinks to the corresponding Pfam family description. Further information about the functional profile can be obtained on two additional results pages. The first page shows a more detailed view on the UFO assignments (see Figure 3): in a scrollable window, for each input sequence (fasta header) the Pfam domain assignments are displayed together with the associated Gene Ontology [19] annotation. While the name of the Pfam and GO categories is shown directly, the corresponding identifiers provide hyperlinks to a detailed description of the associated categories. In addition, the output probability score of the neural network is shown for the particular assignment. The value is in the range between 0.5 and 1.0 with high values above 0.9 usually indicating good matches. On another page the 10 nearest species in terms of the profile divergence with respect to a collection of precomputed reference profiles are shown in a sorted list (see Figure 4). The genome identifiers in that list provide hyperlinks to the corresponding HAMAP description for further information about the associated species. The complete list of 821 reference species together with the corresponding profile divergences (in ascending order) can be obtained as a text file by some link at the bottom of that page.

In the "Downloads" section of the main results page, several output files are available in plain ascii format. In addition a Perl script "ufo2hmmer" for postprocessing of the UFO assignments by means of selected HMMER/Pfam searches can be obtained. This script requires local HMMER and Perl installations and can be used to further increase the specificity of the domain detection. In addition, postprocessing of UFO matches with HMMER provides additional information about sequence positions, repetitions, and the order of the domains.

### Output files

The first output file contains the complete Pfam profile in terms of domain specific detection counts sorted in descending order. The second file contains the corresponding GO profile which shows the assignment frequencies with respect to Gene Ontology categories. The GO counts result from applying the Pfam2GO mapping to the Pfam profile and again the frequencies are shown in descending order. The third file contains the sequence specific assignments to Pfam domain families together with the corresponding GO annotation and the match probability score of the neural network. This file may also be used for further processing, e.g. for Pfam/HMMER search of the UFO detected domains using the provided "ufo2hmmer" script.

### Performance analysis

**Figure 5:**
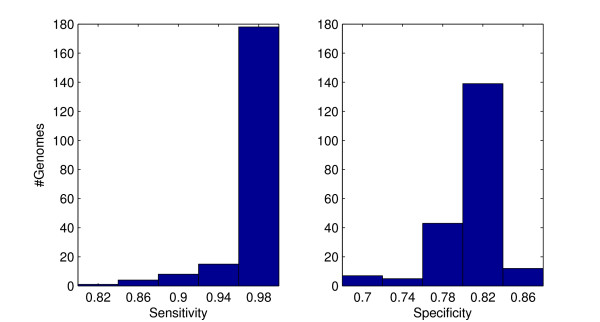
**UFO domain detection performance**. Histograms of UFO protein domain detection sensitivity and specificity.

**Figure 6:**
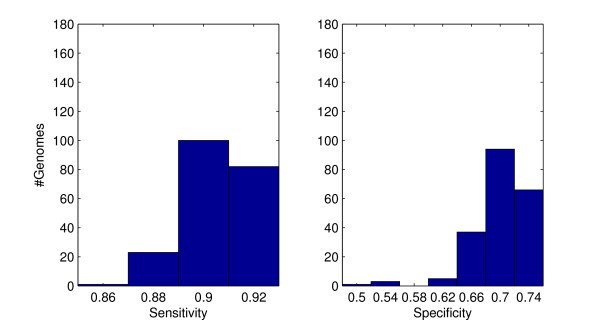
**RPS-BLAST domain detection performance**. Histograms of RPS-BLAST protein domain detection sensitivity and specificity.

The performance of UFO was evaluated in two aspects: first, the accuracy of profiling was measured in terms of the underlying domain detection sensitivity and specificity on whole genome protein collections. Secondly, the speed of UFO was measured in terms of the run time required for the profiling of proteomes. To avoid direct overlap with the training sequences from the Pfam 23 (July 2008) release, I used the (multiple fasta) protein sequence files of 206 genomes from the "latest species" section of the Integr8 web site [20] where these genomes have been included since release 90/91 from January/February 2009. For a complete list of the test genomes see Additional file 1. Sensitivity and specificity were measured by means of a comparison with the available InterPro [21] Pfam hits from the Integr8 ftp site. Considering a single protein sequence, a true positive (TP) is counted if a protein domain family that has been detected by UFO is among the InterPro reference hits. A false negative (FN) occurs if the domain of a reference hit is overseen by UFO and a false positive (FP) is counted if an UFO detected domain family does not occur in the corresponding InterPro reference. For each of the 206 genomes the sensitivity is estimated by #TP/(#TP+#FN) and the specificity by #TP/(#TP+#FP). The mean (median) sensitivity and specificity over all genomes is 97.7 (98.8) and 81.1 (81.8), respectively. The histograms in Figure 5 show that by far in most cases the sensitivity is above 95% with a slightly worse distribution of the specificity. However, because of the high sensitivity, UFO is well suitable for prefiltering. By selective postprocessing with a Pfam/HMMER search for the UFO detected domains (see "ufo2hmmer" in download section) a 100% specificity according to the above definition can easily be achieved. With an average number of 3750 genes, UFO profiling of all 206 genomes took about half an hour corresponding to an average runtime of 9.7 seconds per genome. For comparison the RPS-BLAST tool with the conserved domains database [9], which is widely used for accelerated protein domain searches, was locally installed and applied to the 206 test proteomes. All Pfam 23 domain searches were performed with default parameters and an E-value threshold of 0.001. Comparing the results with the InterPro reference, RPS-BLAST showed a mean (median) sensitivity of 90.6 (90.7) percent and a mean (median) specificity of 69.8 (70.2) percent, which are significantly lower than the corresponding UFO rates. The histograms in Figure 6 show a low variation of the sensitivity but for the specificity some outliers at the lower end increase the range of values. Referring to the runtime, RPS-BLAST took 59 CPU hours for processing of all 206 proteomes, with an average runtime of 17 minutes. Thus, UFO is about 100 times faster than RPS-BLAST.

Table 1 shows a comparison of runtimes for five of the smallest genomes which could also be processed by the batch option at the Pfam web site. The HMMER (version 2.3.2, Oct 2003), RPS-BLAST (version 2.2.16, Mar 2007), and UFO runtimes were measured as (single thread) user times on the same CPU (AMD Opteron 2.0 GHz) showing an UFO speed up of about four and two orders of magnitude if compared to HMMER and RPS-BLAST, respectively. This speed up makes it possible to pass UFO results directly to the user, whereas the Pfam web server (March 2009) with an average processing time of more than 8 hours for the small genomes sends the results by email.

**Table 1:** Runtime comparison for five small genomes between HMMER, RPS-BLAST, UFO and batch search at the Pfam web site (March 2009).

		**CPU time**			
**Species**	**# Proteins**	**HMMER**	**RPS-BLAST**	**Pfam-web**	**UFO**
A. pseudotrichon.	847	19 h 37 m	4 m 11 s	9 h 28 m	2.5 s
E. chaffeensis	803	17 h 58 m	3 m 13 s	9 h 19 m	2.0 s
U. parvum	577	13 h 54 m	3 m 46 s	7 h 38 m	1.8 s
U. urealyticum	611	14 h 36 m	3 m 42 s	8 h 03 m	1.9 s
W. endosymbiont	746	16 h 49 m	3 m 40 s	8 h 42 m	1.9 s

### Example application and discussion

For an example application the proteome of a novel *Clostridium thermocellum *strain (DSM 4150, Integr8 ID: 32332) from the above collection of 206 test genomes was used to demonstrate the servers capabilities. Specifying the multiple fasta file of protein sequences on the UFO job submission page (see Figure 1) and pressing the "Start UFO" button initiates uploading and subsequent analysis of that file. After the processing of all 2916 sequences which takes about 7 seconds, the results page is generated and displayed. The results are based on 3021 assignments to Pfam domains which have been found in a total number of 2087 sequences. This implies that no domains have been found for 829 sequences. Besides the "assignments" page (see Figure 3) and the output files (hyperlinks) which allow a more detailed analysis of the profile properties, the "top ten" lists provide a brief summary of the most prevalent features. In the example (see Figure 2), the most abundant Pfam family is the "Dockerin type I repeat" PF00404 which has been found in 67 sequences. Clicking the identifier shows the corresponding Pfam description of the family which indicates a key role of that domain in cellulose metabolization. Among the top 10 Pfams, also the "Cellulose binding domain" PF00942 found in 18 sequences indicates the importance of cellulose metabolism. The first entry of the nearest species list (see Figure 4) corresponds to another strain of *C. thermocellum *(ATCC 27405/DSM 1237) with a slightly bigger proteome set including 3102 proteins. According to the corresponding HAMAP description (hyperlink), *C. thermocellum *is a gram-positive, anaerobic, and thermophilic organism capable of cellulose degradation. The remaining species in the list also belong to the class of Clostridia, most of them are thermophilic. The closest five species are all able to ferment organic substrates.

As indicated by the application example above, the strength of UFO is its capability to produce a quick overview on the functional inventory of whole genomes in terms of the most abundant protein domains and in terms of the closest organisms with the most similar profiles. In comparison to full annotation servers like RAST [22] the UFO server only covers a particular aspect of genome annotation. It merely provides a first step of a functional analysis which can nevertheless be of great utility for addressing many biological questions and problems. It is not restricted to the analysis of prokaryotic genomes, and it can be applied to eukaryotes as well, if gene predictions are available. The runtimes for complete eukaryotic proteomes are usually above the average runtime for prokaryotes. For example, the proteome set of *D. melanogaster *(15410 sequences) takes 64 seconds of processing time, the *C. elegans *proteome (22984 sequences) requires 80 seconds. In addition, UFO can also be used to annotate large collections of (translated) expressed sequence tags. For prokaryotes the UFO domain detection can be used as a basis for the prediction of operons or regulons. Furthermore, the server supports researchers in the identification of functionally related species that can be used for annotation. For the analysis of microbial communities, gene prediction tools specialized on short anonymous DNA fragments [13,23,24] or a simple six-frame translation can be used to apply UFO in functional metagenomics. In comparison to the more comprehensive MG-RAST server [25], UFO provides an easy-to-use interface with immediate response. For example, UFO profiling of the first of ten depth-specific data sets from the hypersaline microbial mat metagenome [26], which contains 12218 sequencing reads with an average length of 700 bp, requires 75 seconds for processing of the six-frame translated reads. The processing of all ten data sets takes about 15 minutes. Inspection of the top ten Pfams shows a remarkable count for sulfatase (PF00884) assignments in lower layers with a maximum of 135 assignments in the fifth layer (4-5 mm depth), which is in accordance with the results in [26]. In general, the profile divergence with respect to the reference genomes is of limited use for metagenome analysis because a metagenomic profile actually corresponds to a mixture of several different species. However, if the habitat is dominated by a few closely related species, the UFO "top 10 nearest species" list may nevertheless be informative. In case of the hypersaline microbial mat the UFO results indicate a dominant role of Cyanobacteria in the two upmost layers (0-1 mm and 1-2 mm) with 9 and 6 out of 10 nearest species, respectively. This observation is in agreement with the analysis in [26] which indicates that Cyanobacteria together with Alphaproteobacteria are the most abundant phyla in these layers. Especially in metagenomics, the GO profile may facilitate the analysis because Pfam assignments are accumulated in categories, which directly relate to the biological questions. For example, considering the frequencies of the "chemotaxis" term (GO:0006935) for the hypersaline microbial mat data, the maximum count (55 assignments) is found in the third layer (2-3 mm) at the oxic-anoxic boundary, which agrees well with the original study [26].

## Conclusion

With a considerable speed up of protein domain detection, UFO shows a new perspective in web-based large scale analysis of protein sequence data. As a consequence of its speed it can be used for instantaneous profiling of genome scale protein sequence files. The processing time roughly corresponds to the duration of a single sequence analysis as provided by current protein database servers. On the scale of prokaryotic genomes, UFO can process thousands of whole genome protein sets a day. In that way UFO is well prepared for next generation sequencing technologies like single cell sequencing [27], which allows to extract whole genomes from highly diverse metagenomic samples.

## Availability and requirements

The UFO web service is freely available at .

## Authors' contributions

PM designed the database interface and the search engine and wrote the manuscript. The author read and approved the final manuscript.

## Supplementary Material

Additional file 1 **Test genomes used for performance evaluation**. The file shows a list of all 206 test genomes that were used for evaluation of the UFO web server. The complete proteomes were obtained from recent Integr8 data base updates and correspond to all entries of the "latest species" section which were added in release 90/91 from January/February 2009. Click here for file
