# Supplementary material for: UFO: a web server for ultra-fast functional profiling of whole genome protein sequences
Source: BMC Genomics. 2009 Sep 2;10:409. doi: 10.1186/1471-2164-10-409 (PMC2744726; doi:10.1186/1471-2164-10-409)
Supplement: Additional file 1 — Test genomes used for performance evaluation. The file shows a list of all 206 test genomes that were used for evaluation of the UFO web server. The complete proteomes were obtained from recent Integr8 data base updates and correspond to all entries of the "latest species" section which were added in release 90/91 from January/February 2009. [file 1471-2164-10-409-S1.pdf]

UFO: a web server for ultra-fast functional profiling of whole genome protein sequences
Supplementary material

Peter Meinicke

The test genomes for evaluation of the UFO web server were obtained from recent Integr8 data base updates and correspond to all entries of the “latest species” section which were added in release 90/91 from January/February 2009. A complete listing of the 206 entries is given below.

```
31898.P_chrysogenum
32114.P_berghei
31998.P_chabaudi
32557.B_catenulatum
32188.S_agalactiae_COH1
32184.B_sp_SG-1
32182.B_sp_H160
32180.S_pneumoniae_SP9-BS68
31894.A_pseudotrichonymphae
32178.B_anthracis_Tsiankovskii-I
32176.B_thuringiensis_ATCC_35646_israelensis
32174.V_shilonii
32168.O_indolifex
32166.L_sp_PCC_8106
32164.B_graminis
32162.P_sp_BAL39
32160.B_animalis
32158.L_araneosa
32154.H_parasuis
32152.H_sp
32148.Y_pestis
32146.E_coli_O157-H7_EC4401
32144.M_marina
32142.X_fastidiosa_Dixon
32140.U_parvum
32138.R_sp
32134.L_monocytogenes_F6854
32132.B_sp_SS
```

32130.R_bacterium
31846.G_diazotrophicus
32128.S_agalactiae_18RS21
32126.E_coli_O157-H7_EC4501
32124.C_perfringens_C_JGS1495
32122.T_carboxydivorans
32120.C_jejuni_CG8486
32118.C_perfringens_B_3626_ATCC
32116.H_influenzae_221-21
32110.E_coli_O157-H7_EC508
32108.R_sp_SK209-2-6
32106.H_influenzae_R3021
32104.V_cholerae_2740-80
32102.P_maris
32100.B_cereus_AH1134
31810.T_onnurineus
31802.C_burnetii_Q154
31800.C_burnetii_Q212
32298.C_perfringens_CPE_F4969
32296.S_pneumoniae_SP3-BS71
32294.H_influenzae_224-21
32292.B_anthracis
32290.S_pneumoniae_SP195
32288.S_enterica_SARA29
32286.B_anthracis_A0193
32284.W_endosymbiont
32282.E_coli_O157-H7_EC4196
32280.M_sp_PE36
31994.C_perfringens_E_JGS1987
31992.C_aerofaciens
31990.S_enterica_191
32278.A_bacterium_TW-7
32276.B_pseudomallei_305
32274.C_burnetii_RSA_334
32272.V_bacterium
31986.S_enterica_SARA23
31984.S_enterica_SL480
31982.D_sp
31980.S_pneumoniae_SP11-BS70
32268.E_coli_O157-H7_EC869
32266.d_proteobacterium
32264.B_anthracis_A0174
32262.S_enterica_HI_N05-537
32260.C_flavus
31976.u_eubacterium
31974.N_spumigena

31972.F_bacterium
31970.C_perfringens_D_JGS1721
32258.B_cereus_NVH0597-99
32254.G_sp_G11MC16
32252.G_sp
31968.C_sp_PCC_7822
31966.R_pickettii
31964.S_pneumoniae_SP23-BS72
31962.D_acetoxidans
31960.C_jejuni_84-25
32248.S_pneumoniae_SP19-BS75
32246.L_reuteri_100-23
32242.U_parvum_2783_ATCC
31958.Y_pestis_B42003004
32240.S_agalactiae
31954.C_jejuni
31952.S_agalactiae_CJB111
31950.V_cholerae_MAK_757
32238.B_cereus_03BB108
32236.M_sp
32234.W_endosymbiont_of_Drosophila_ananassae
32232.V_cholerae_V52
31948.M_luteus
32230.U_urealyticum_33698_13
31944.B_sp_PS
31942.U_urealyticum_5
31940.E_coli_O157-H7_EC4486
32228.E_chaffeensis_Sapulpa
32224.H_influenzae_PittII
32222.V_cholerae
31938.S_putrefaciens_200
32220.E_coli_O157-H7_EC4076
31936.R_sibirica
31934.C_butyricum
31932.S_stellata
32218.S_pneumoniae
32216.S_pneumoniae_SP14-BS69
31928.R_sp_CCS2
32210.C_mediatlanticus
31926.S_enterica_SL486
31924.Y_pestis_MG05-1020
31922.S_benthica
31920.E_coli_F11
32208.C_watsonii
32206.C_jejuni_26094
32204.m_gamma_proteobacterium_HTCC2143

32202.T_bacterium
31918.S_enterica_SL317
32200.S_pneumoniae_SP6-BS73
31916.B_caccae
31912.R_sp_TM1035
31910.E_faecium
31902.L_sp
31900.L_sp_Group_II_5-way_CG
32098.E_sp_SD-21
32096.S_agalactiae_515
32094.B_anthracis_A0442
32092.B_anthracis_A0465
32088.U_urealyticum_11
32086.B_ambifaria_IOP40-10
32084.A_sp
32082.S_pneumoniae_CDC1087-00
32080.R_grylli
31795.R_centenum
31793.H_pylori_P12
31791.E_coli_SE11
32076.M_voltae
32072.V_campbellii
32070.V_vadensis
32068.G_sp_M21
32066.C_botulinum_Bf
32064.R_solanacearum
32060.C_jejuni_CG8421
32058.B_cereus_G9241
32056.C_sp_AT7
32054.S_enterica_RI_05P066
32052.E_coli_O157-H7_EC4113
32050.R_leguminosarum
32048.V_cholerae_MZO-3
32046.C_sp_CCY_0110
32044.P_pacifica
32042.S_enterica_4-5-12-i--_CVM23701
32040.L_monocytogenes_H7858
32038.C_perfringens_NCTC_8239
32034.E_coli_E22
32032.L_aggregata
32030.P_sp
32028.F_nucleatum_ATCC_49256_vincentii
32026.H_influenzae_PittAA
32022.A_maxima
32018.Y_pestis_F1991016
32016.E_sp_AT1b

32014.S_dysenteriae_1012
32012.H_influenzae_3655
32010.M_algicola
32008.C_botulinum
32006.a_proteobacterium
32004.B_coagulans
32002.S_pneumoniae_CDC3059-06
32000.C_coli
32356.U_urealyticum_33696
32354.E_coli_101-1
32352.Y_pestis_UG05-0454
32350.B_ambifaria
32348.B_anthracis_A0389
32346.H_influenzae_PittHH
32344.C_sp_PCC_8802
32342.B_cereus_W
32338.C_botulinum_NCTC_2916
32336.X_fastidiosa_Ann-1
32334.Y_pestis_E1979001
32332.C_thermocellum
32330.S_enterica
32328.B_selenitireducens
32326.U_parvum_14
32324.E_coli_B7A
32322.S_pneumoniae_MLV-016
32320.C_jejuni_CF93-6
32318.E_coli_E110019
32316.O_bacterium
32314.C_ferrooxidans
32312.U_urealyticum
32310.F_bacterium_ALC-1
32308.S_pneumoniae_CDC1873-00
32306.U_urealyticum_33175
32304.B_sp
32302.L_sp_MED105
32300.A_vinelandii
32198.m_gamma
32196.C_upsaliensis
32194.S_pneumoniae_CDC0288-04
32192.G_sp_Y412MC10
